# Glances at the Hospitals

**Published:** 1901-11-02

**Authors:** 


### GLANCES AT THE HOSPITALS.

### PETERBOROUGH INFIRMARY.

The total income for the year amounted to £2,097, and the total expenditure to £2,096. We notice that patients using the private wards paid £133 during the year, and that "patients' shillings" amounted to £18 10s. The governors refer to the necessity of doing something to the men's medical ward to make it more sanitary and more commodious, it being the only part of the hospital requiring attention. The total number of in-patients was 492, and of out-patients 3,292, making a grand total of 3,784. Of these 2,926 were cured, 282 relieved, 12 incurable, 58 died, and the rest were discharged for various reasons. The tables are properly drawn out, except that it would be better to give the results of the in- and out-patients in separate columns. We notice that of 15 cases of pneumonia treated during the year 13 recovered and 2 died; there were 11 cases of enteric fever and all recovered; and of 48 cases of acute rheumatism only 1 died. Of 19 operations for cataract all were successful, and so with 5 cases of ovariotomy. The anæsthetic table shows that ether was used 105 times, chloroform 83, and gas 17.

### ARMAGH COUNTY HOSPITAL.

The total receipts amounted to £1,386, and this sum included "county presentments" of £894. The expenditure was £1,504, and there was a balance due to the bank of £214. The hospital contained 37 patients on January 1st, and 462 were admitted during the year. Of these 453 were discharged cured, relieved, or incurable, and 12 died. Tables II. and III. give an analysis of the more important cases treated, and of the principal operations performed during the year. Unfortunately the first of these tables gives numbers only, but the second shows results as well. There is also a table showing the causes of death.

### LEICESTER INFIRMARY.

The total income from all sources was £16,514, and the total ordinary expenditure was £12,938. About £666 was spent on the building account, and a balance of £4,163 was transferred to the fever house. The workpeople's donations reached £3,545. The governors say that "the large and increasing number and serious nature of the operations performed in the infirmary have rendered the present theatre utterly inadequate for its purpose," and it is proposed to erect a new wing which will contain two operating theatres, a casualty room, and various other offices. The cost is anticipated to be about £10,000. The in-patients numbered 2,799 during the year, and the out-patients 17,644. The daily average number resident in the infirmary was 184, and the average stay in the wards was 24 days. The average cost of each in-patient was £3 17s. 2d., a sum which exceeds that of 1899 by 5s. 4d. The average cost of each out-patient was 2s. 4¾d. As already said, the total number of in-patients was 2,799. Of these 2,320 were treated in the infirmary proper, 396 in the children's hospital, and 83 in the fever house. Of the infirmary cases, 1,616 were discharged cured or relieved, unrelieved 21, for other reasons 60, to convalescent home 36, made out-patients 291, died 152, and 144 remained under treatment. At the children's hospital 254 were discharged cured or relieved, 46 were made out-patients, 28 for various reasons, 43 died, and 25 remained under treatment. At the fever house 62 were discharged cured, eight were sent to convalescent house, four died, and nine remained under treatment. So far the statistics are fully given in the report, and they are easily followed, but we regret that we cannot say the same of the medical and surgical records. Of medical statistics there are none printed. There is a list of operations which tells us that 686 major operations, and 1,134 minor operations were performed, and there is also a list of the more important accidents, but in neither case are the results shown. It is not much use to tell us that trephining the skull was performed 9 times if we are not also told the object of the operation, and whether that object was attained or not. In the ophthalmic department 198 operations were performed, and in the ear and throat department 1,130 patients were treated, but there is the same lack of information about them. In the Roentgen ray department 229 cases presented themselves for investigation, and the apparatus has worked uniformly well. We are pleased to notice a table showing the nature of the anæsthetics used in the operations. Chloroform was the agent in 894 instances; ether in 689; gas in 167, and a mixture of chloroform and ether in 70.

### LAKESIDE HOSPITAL, CLEVELAND, OHIO.

Judging from the reports of this institution it would seem that the infirmaries of the United States are, like those of our own country, not always in a flourishing financial condition. In this hospital there was a deficit at the end of the year of $17,786. There are separate wards for private patients, and the daily average number of patients in these rooms was 33, the daily average number resident of all classes being 135. In addition to a large visiting staff of physicians and surgeons, there are 16 resident medical officers distributed as follows:—Four in medical wards, 5 in surgical, 4 in gynæcological, 3 in the private wards, and 1 pathologist. The pathological department is a detached block of two stories, and a third story is about to be added, for which a generous donor, Mr. Hartness Brown, has given $5,000. The medical and surgical tables are well arranged, showing the numbers affected with the various diseases, the numbers cured, not cured, and died. There were, for instance, 8 cases of diphtheria, and all recovered. Of pleurisy there were 14 cases. Of these 7 recovered, 5 improved, 1 not improved, and 1 not treated. There were 139 cases of typhoid fever, and of these 128 recovered, 5 died. There were 13 cases of cataract. Of these 9 were cured and 4 improved. The surgical report shows that in 100 consecutive cases of abdominal section there was no death. Among the 100 the appendix was removed 44 times. Altogether there have been in two years 233 cases of abdominal section with 3 deaths.

### STOCKTON AND THORNABY HOSPITAL.

The income amounted £3,080, and the expenditure to £2,761. There was a balance of £1,538 in favour of the hospital at the beginning of the year and at the end of the year that balance had risen to £1,858. The workmen's subscriptions were £2,310, being an increase of £139 on the previous year. The finances are in a sound condition. The total number of in-door patients was 610, and that of the out-door patients was 1,640. The average stay of each patient in the hospital was 20½ days, and the cost per day was 4s. 5d. The operation table shows the numbers of patients operated on and the results; but the other surgical tables and the medical tables merely give the numbers suffering from the various diseases.

### CHESTER GENERAL INFIRMARY.

The total income was £4,820, and the total ordinary expenditure was £4,952. The year closed with a balance of £881 due to the treasurer. The infirmary contained 91 patients at the beginning of the year, and 1,168 were admitted during it, making a total of 1,259 under treatment. Of these 694 were cured, 367 relieved, 29 not relieved, 2 discharged for special reasons, 75 died, and 92 remained in the infirmary. The mortality was 5·4 per cent. The highest number resident was reached on November 27th when 105 were in the house. There were 4,493 out-patients. The medical and surgical tables are carefully drawn out. There were 34 amputations with 2 deaths, and three excisions of the hip-joint, 2 being cured and one relieved. Ovariotomy was performed 5 times, all being successful. There is a table showing the causes of death in the 75 cases, but no anæthstic table is given.

